# A Wireless Implantable System for Facilitating Gastrointestinal Motility

**DOI:** 10.3390/mi10080525

**Published:** 2019-08-09

**Authors:** Po-Min Wang, Genia Dubrovsky, James C.Y. Dunn, Yi-Kai Lo, Wentai Liu

**Affiliations:** 1 Department of Bioengineering, University of California, Los Angeles, Los Angeles, CA 90095, USA; 2 Division of Pediatric Surgery, Department of Surgery, David Geffen School of Medicine, University of California, Los Angeles, Los Angeles, CA 90095, USA; 3 Division of Pediatric Surgery, Department of Surgery, Stanford University School of Medicine, Stanford, CA 94305, USA; 4 Department of Bioengineering, Stanford University, Stanford, CA 94305, USA; 5 Niche Biomedical Inc., Los Angeles, CA 90095, USA

**Keywords:** gastrointestinal stimulation, implant, motility, neuromodulation, bioelectronics medicine, electroceuticals

## Abstract

Gastrointestinal (GI) electrical stimulation has been shown in several studies to be a potential treatment option for GI motility disorders. Despite the promising preliminary research progress, however, its clinical applicability and usability are still unknown and limited due to the lack of a miniaturized versatile implantable stimulator supporting the investigation of effective stimulation patterns for facilitating GI dysmotility. In this paper, we present a wireless implantable GI modulation system to fill this technology gap. The system consists of a wireless extraluminal gastrointestinal modulation device (EGMD) performing GI electrical stimulation, and a rendezvous device (RD) and a custom-made graphical user interface (GUI) outside the body to wirelessly power and configure the EGMD to provide the desired stimuli for modulating GI smooth muscle activities. The system prototype was validated in bench-top and in vivo tests. The GI modulation system demonstrated its potential for facilitating intestinal transit in the preliminary in vivo chronic study using porcine models.

## 1. Introduction

The estimated annual healthcare expenditures for gastrointestinal (GI) motility disorders is USD 29 billion, which imposes a significant burden on the U.S. healthcare system [1]. GI motility disorder is defined as the abnormal movement behavior of the GI tract that affects the function of mixing and propelling food. This can happen to any segment of the GI tract, including the esophagus (e.g., dysphagia, achalasia), stomach (e.g., gastroparesis, gastroesophageal reflux disease), and intestines (e.g., diarrhea, constipation). The pathophysiological mechanism of GI motility disorders is not completely understood due to the complex, cooperative mechanisms between the smooth muscle cells (SMC), interstitial cells of Cajal (ICC), enteric nervous system, central nervous system, and hormones [2,3]. This gap of knowledge impedes the development of efficacious therapies for improving GI motility. Although a number of pharmaceutical drugs have been developed, most of them do not completely alleviate GI dysmotility [4]. Moreover, the use of drugs faces the challenge of target specificity, as it is difficult to precisely control the dose of pharmacological agents. 

A potential treatment alternative for GI motility disorders is GI electrical stimulation. The fundamental principle behind this therapy is to electrically modulate myoelectric activity that controls smooth muscle contraction/relaxation, i.e., slow waves and spikes [5]. A slow wave is a rhythmical electrical event originating from ICC, while a spike is the action potential that stems from the inflow of calcium ions to the SMC. ICC and SMC are both regulated by the enteric nervous system in the GI tract [6,7]. It is thus feasible to directly induce smooth muscle contraction through modulating SMC or indirectly via activating the ICC/myenteric network using GI electrical stimulation. Several studies have demonstrated the therapeutic potential of GI electrical stimulation in GI dysmotility, including gastric electrical stimulation for pharmaceutically intractable gastroparesis [8,9], intestinal electrical stimulation for accelerating intestinal transit [10,11], and colonic electrical stimulation for constipation [12,13]. The potential mechanisms are either the stimulation of the SMC [8,9] or the activation of the cholinergic or nitrergic pathways in the enteric nervous system [10,11,12,13].

Despite promising research results, the clinical applicability and usability of GI electrical stimulation for GI motility disorders is still limited due to the lack of appropriate implantable stimulation devices. In light of the fact that existing commercial implantable pulse generators (e.g., deep brain stimulator, spinal cord implant, vagus nerve stimulator [14,15,16]) have limited parameter programmability, the implantable GI stimulator should support a wide range of stimulation parameters (i.e., pulse width, current intensity, frequency), especially long-pulse stimuli to activate smooth muscle when necessary. Interestingly, even on the same GI segment, diverse stimulation parameters have been reported for improving GI dysmotility, e.g., colonic transit could be accelerated in rats by stimulation protocols of 4 ms, 10 mA, 40 Hz [17], and 0.3 ms, 5 mA, 10 Hz [18]. It is thus essential to develop a device capable of versatile stimulation parameters in order to customize an optimal stimulation strategy for each individual. This above feature is also critical, as the investigation of an optimal stimulation protocol for effectively activating GI contraction and the sophisticated working mechanism of the GI tract is still ongoing. Another desired feature for GI implants is the miniaturized form factor. This would be clinically beneficial, as surgical invasiveness can be minimized during implantation. Existing commercial GI implants are bulky and only support stimuli with a very limited range of parameters, e.g., pulse width shorter than 1 ms [19,20]. The short pulse width limits their usage in treating nausea and vomiting [19] or increasing the pressure of lower esophageal sphincter for relieving gastroesophageal reflux disease [20]. Both commercial devices might not be applicable to directly altering GI motility as most protocols for inducing GI contraction require a stimulation pulse greater than 1 ms [8,9,10,11,12,13]. In order to fill the technology gap, a miniaturized implantable GI stimulator supporting a wide range of stimulation parameters is needed.

**Figure 1:**
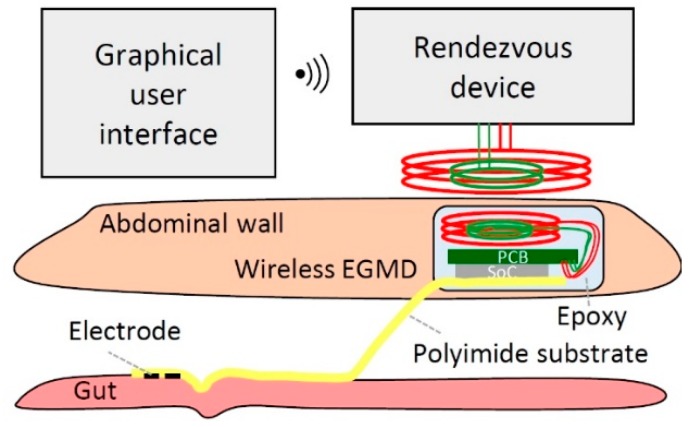
Illustration of wireless gastrointestinal (GI) modulation system interfacing with gut.

We developed a wireless GI modulation system that fulfills the aforementioned needs (Figure 1). The system consists of a wireless extraluminal gastrointestinal modulation device (EGMD) interfacing with the gut, a custom-made graphical user interface (GUI) on a tablet for configuring the EGMD, and a rendezvous device (RD) serving as a relay between the EGMD and the GUI. The core of the EGMD is a system on a chip (SoC) capable of generating versatile stimulation patterns through GUI control to modulate GI motility and wireless power and data transfer via inductive links. A heterogeneous packaging approach integrates the SoC and printed circuit board (PCB) with a polyimide-based electrode array, which achieves device miniaturization. The EGMD is encapsulated by biocompatible epoxy and implanted in the abdominal wall through midline abdominal incision. The RD carried by the subject receives user-defined commands from the GUI via WiFi link and delivers power and command to configure the EGMD through inductive links. Design consideration and the implementation of the EGMD were presented, and preliminary in vivo acute tests were performed [21]. Building upon our prior work, the EGMD was advanced to a fully implantable GI modulation system; a preliminary in vivo chronic study was conducted using porcine models to investigate its potential for accelerating intestinal transit.

The remainder of this paper is organized as follows. Section 2 presents the microelectronic design of the wireless implantable GI modulation system and the heterogeneous packaging technique that achieves implant miniaturization. Section 3 presents experiment results of the GI modulation system in bench-top and in vivo chronic tests. The conclusion is drawn in Section 4.

## 2. Wireless Implantable GI Modulation System

### 2.1. Microelectronic Design

**Figure 2:**
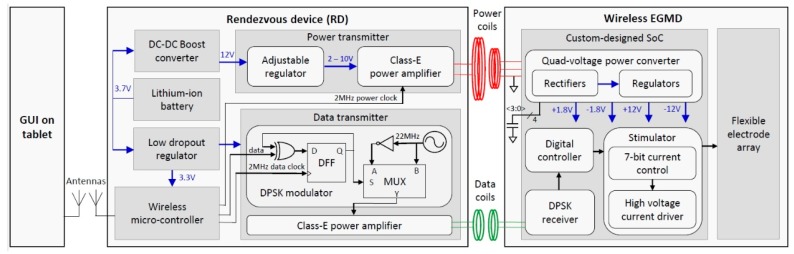
Functional block diagram of wireless GI modulation system.

The custom-designed SoC in the wireless EGMD adapted from our prior work supports wireless power and data transfer, and highly programmable current-mode stimulation for activating GI contraction (Figure 2) [22]. The quad-voltage power converter consisting of rectifiers and voltage regulators receives inductive power signals from the RD and provides regulated +/–1.8 V to supply the data receiver and the control logic, and +/–12 V to the current driver in the stimulator. The high compliance voltage for the stimulator is critical as it allows a wide range of parameters for current-mode stimulation. The differential phase shift keying (DPSK) receiver that is immune to the interference in the amplitude domain demodulates the incoming DPSK signal back to the digital command issued by the user. The command is then decoded by the digital controller, and the stimulator is set to accordingly generate the desired electrical stimuli. The highly programmable digital controller enables the stimulator to support intensities from 0.3 μA to 20 mA, with a pulse width from 10 μs to a user-defined length, and a frequency from 0.001 to 200 Hz. 

The RD made by off-the-shelf components receives the command from the GUI on the tablet via WiFi and, accordingly, powers and configures the EGMD through dual-band inductive telemetries. Carrier frequencies of the power and data links are 2 and 22 MHz, respectively. This frequency separation, one decade apart, allows the use of signal filtering in the DPSK receiver to relieve power to data interference. In the power transmitter, a highly efficient class-E power amplifier was designed to drive the primary-side power coil [23]. The power amplifier is supplied by an adjustable regulator with voltage ranging from 2 to 10 V. This adjustability enables the power transmitter to tune the output-power level based on the variation of coupling coefficients between coil pairs in order to optimize power-transfer efficiency. On the other hand, in the data transmitter, the same architecture of class-E power amplifier is used, and the amplifier is driven by a noncoherent DPSK modulator [24]. This modulation scheme encodes the command bit in the relative phase shift between two consecutive symbols rather than the phase shift to a reference clock, which avoids a complicated synchronization circuit in the implant side, e.g., phase-locked loop. The scheme is realized by a XOR gate and a D flip-flop, such that the D flip-flop toggles when the input bit is logic one and remains unchanged when the input bit is logic zero (Figure 2). The clocks and data command required by the power and data transmitters are generated by a microcontroller (CC3200, Texas Instruments., Dallas, TX, USA) that can be wirelessly controlled by the tablet through WiFi. The DC–DC converter and low-dropout regulators take input from a 3.7 V lithium ion rechargeable battery to power circuits in the RD. The wireless and battery-power features allow the RD to be carried by freely moving subjects receiving the implant.

### 2.2. Heterogeneous Packaging and System Integration

**Figure 3:**
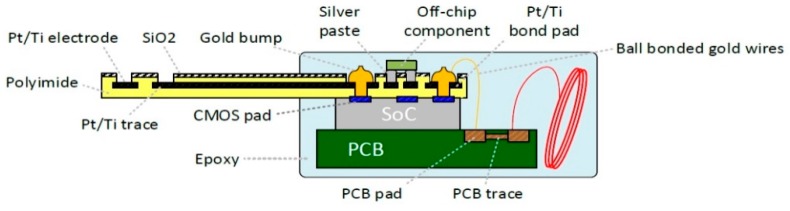
Illustration of heterogeneous packaging for wireless extraluminal gastrointestinal modulation device (EGMD).

The heterogeneous-packaging technique utilizes an 8 μm thick polyimide substrate to integrate the SoC, PCB, off-chip components, and electrode array into a miniaturized EGMD (Figure 3). The electrodes, bond pads, and the interconnections on the substrate are made of platinum/titanium (200/10 nm thickness) fabricated by the e-beam-evaporated deposition technique [25]. All required electrical connections for enabling system functions (e.g., the connection between electrodes and outputs of stimulation drivers) are achieved by the patterned metal trace on the polyimide substrate, as well as the gold bumps and ball-bonded gold wires. The gold bumps connect the polyimide substrate with the SoC, while the connection among the substrate, SoC, and the PCB are made by the ball-bonded gold wires. The use of a small-footprint PCB significantly facilitated the development of the EGMD prototype. This configuration enables design debugging and the monitoring of critical signals in the SoC by directly probing PCB pads during the system bench top test. Nevertheless, it should be noted that the PCB is not a necessary part in the final EGMD design. Indeed, all off-chip components can be connected to pads on the polyimide substrate via the silver conductive paste in the absence of a PCB. After the assembly process, the EGMD is encapsulated by the epoxy (3M DP100 Plus Clear, 3M Corp., Maplewood, MN, USA). The materials interfacing with the biological tissue, including the polyimide, platinum, and epoxy, are all biocompatible. 

**Figure 4:**
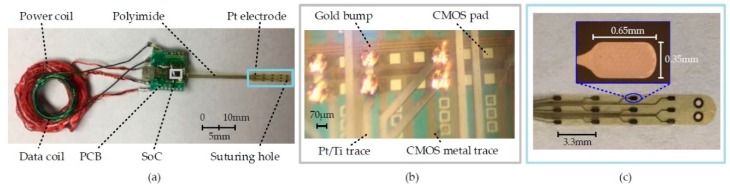
(**a**) Photo of EGMD prototype for chronic study. Zoom-in view of (**b**) system on a chip (SoC) part under microscope and (**c**) electrode array.

Figure 4 shows the miniaturized EGMD prototype. The coaxial arrangement of power and data coils reduces EGMD size. The separation between the coils and the PCB were suggested by the surgeon in favor of EGMD implantation during the surgical procedure. Suturing holes were designed on the tip of the electrode array for anchoring the array on the gut by suture. The miniaturized EGMD has a weight of ~4.2 g and a volume of ~4.9 cm^3^, which is at least an order lighter and 7.7 times smaller than existing commercialized GI implantable stimulators [19,20].

## 3. Experiment Results and Discussion

The proposed wireless implantable GI modulation system was used to study its potential to improve intestinal motility in porcine models. In order to ensure effective and safe intestinal stimulation, the experiment protocol was designed as follows: 1) The charge injection limit and impedance of the electrode–tissue interface were first characterized using cyclic voltammetry (CV) and two different impedance-measurement methods. This provides a guideline for a safe stimulation protocol that avoids any electrochemical tissue-damaging reaction in the electrode–tissue interface [26]. 2) An acute in vivo test using the electrode array was then performed to investigate the stimulation protocol that is also electrochemically safe for facilitating intestinal motility. 3) The wireless GI modulation system was tested on the bench top to ensure its capability of generating the desired stimulation pattern identified in the acute test. 4) A preliminary in vivo chronic test was finally conducted to demonstrate the wireless GI modulation system as an effective tool for accelerating intestinal transit in porcine models. The use of all animals was approved by the UCLA Animal Research Committee (Institutional Review Board no. 2014-142-03E).

### 3.1. Electrode Characterization

The electrode–saline interface and electrode–intestinal fluid interface were both characterized for stimulation-safety evaluation, as both extraluminal and intraluminal intestinal stimulation have been reported as potential treatments for intestinal dysmotility [10,27]. A 0.1 M NaCl solution was used to mimic the extraluminal environment, while intraluminal intestinal fluid was extracted from a juvenile mini Yucatan pig. A customized electrode array that was fabricated by the same process and had the same electrode dimension with the electrode array on the EGMD was soaked separately in both saline and intestinal fluid. The charge injection limit, bioimpedance, and Randles cell model of both electrode–electrolyte interfaces were characterized using CV, electrochemical impedance spectroscopy (EIS), and the time-domain Randles cell model characterization method [28], respectively. 

**Figure 5:**
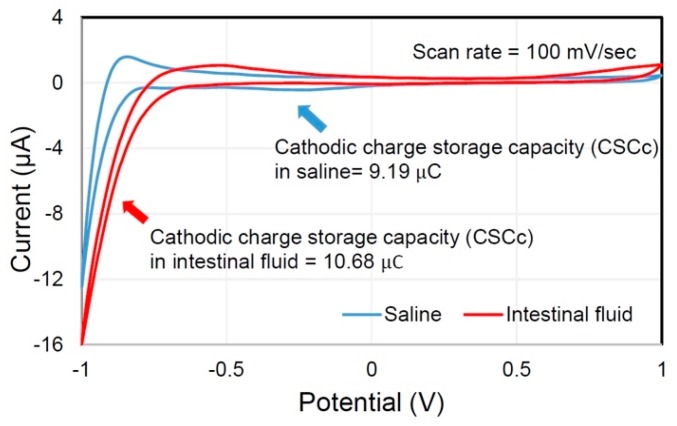
Measured cyclic voltammogram of electrode array in saline and intestinal fluid.

The measured cyclic voltammogram is shown in Figure 5, and all measured electrochemical properties are summarized in Table 1. The electrochemical window of the electrode was [–0.9, 1 V] in both saline and intestinal fluid environments. Charge storage capacity (CSC) of the electrode, which defines the charge injection limit per stimulation phase, of the intestinal fluid group was 1.16 times higher than the saline group (10.68 vs. 9.19 μC). The EIS-measured impedance of the electrode at 1 kHz of the intestinal fluid group was 2.45 times higher than the saline (6.39 vs. 2.61 kΩ), which implies that the maximum allowable stimulation current without exceeding the compliance voltage of the stimulator was ~2.45 times smaller in intestinal fluid than in saline. The measured Randles cell model of the electrode also provided the same insight, as R_S_ was 2.26 times larger in the intestinal-fluid group (3.69 vs. 1.63 kΩ). The product of R_CT_ and C_dl_, i.e., RC time constant, was comparable between the two groups (29.70 vs. 29.11 μs), suggesting that the required time to discharge residual electrical charge at the stimulation electrode was similar. According to this experiment result, extraluminal stimulation is more beneficial compared to intraluminal, as it allows a 2.45-fold higher stimulation current, with an acceptable penalty of 1.16-fold decrease in charge injection limit.

### 3.2. Stimulation-Parameter Identification

**Figure 6:**
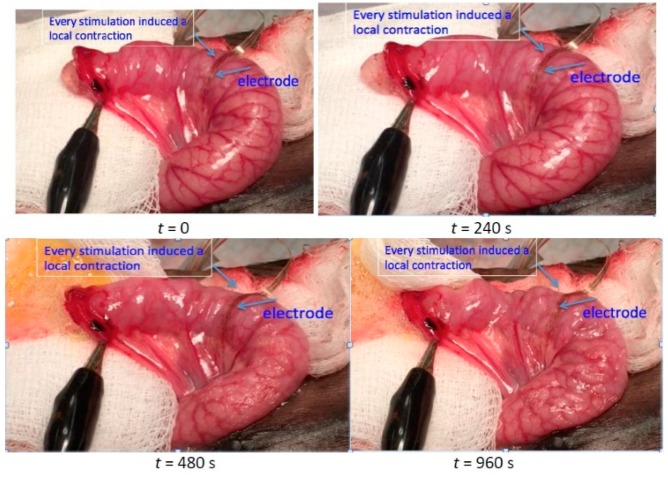
In vivo acute test showing that intestinal local contraction and peristalsis were initiated through the identified stimulation protocol.

In vivo acute tests using juvenile mini Yucatan pigs were conducted to investigate the effective and safe intestinal-stimulation protocol for improving intestinal dysmotility. The animal preparation and surgical procedures were described in our work [11]. During the experiment, a midline laparotomy was performed, and a short segment of jejunum was identified and externalized. The jejunum was transected, and 5 mL of ultrasound gel was placed inside (Figure 6). The segment of jejunum was then monitored for 20 minutes, first under no stimulation, and then under electrical stimulation generated by our SoC via the customized electrode array laid on top of the exposed segment. The gel forced back out of the intestine via peristalsis was collected and weighed for each 20-minute time interval.

Effective stimulation parameters for increasing the rate of peristalsis were investigated in our previous work [11]. One set of biphasic current stimuli (100 Hz, 2 mA, and 2 ms) was obtained and was able to induce both local contraction and peristalsis (Figure 6). The result showed that in a 20-minute time period, an average of 0.51 grams of gel were expelled without intestinal stimulation, while 1.67 grams of gel were expelled with stimulation (*n* = 6, p < 0.05). The potential mechanism is the activation of ICC or the local enteric nervous system by stimulation. The identified stimulation protocol is electrochemically safe in terms of charge injection limit. The stimulus with 2 mA intensity and 2 ms pulse width lead to a 4 μC charge per stimulation phase, which was smaller than the charge-storage capacity of the electrode (9.19 μC) characterized in the CV test. In addition, based on the Randles cell model of the electrode–tissue interface characterized in the impedance measurement, it could be estimated that the peak-to-peak voltage induced by this current stimulation pattern was around +/–7.5 V, which was within the compliance voltage of the stimulator.

### 3.3. System Bench-Top Test

**Figure 7:**
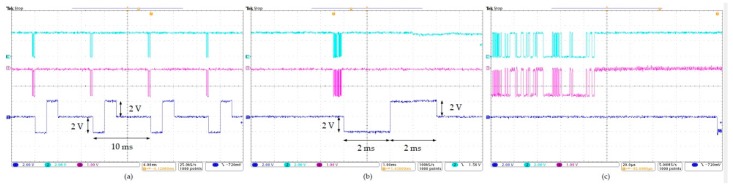
Stimuli of 100 Hz, 2 mA, and 2 ms were generated by the wireless GI modulation system on the bench top. Cyan, pink, and blue traces are command sent from microcontroller unit (MCU) in the RD, command demodulated by differential phase shift keying (DPSK) receiver in EGMD, and the induced output voltage waveform, respectively. Traces presented in time period of (**a**) 40 ms, (**b**) 10 ms, and (**c**) 200 μs.

The wireless GI modulation device was tested on the bench top to verify its capability of generating the desired stimulation protocol. The EGMD was powered and configured by the RD through inductive links with a separation of 2 cm between the primary-and secondary-side coils. A GUI on an Android tablet wirelessly controlled the RD to generate the power and data signals required by the EGMD [29]. The output of the stimulator was connected to a 1 kΩ resistor through PCB pads for current intensity characterization. An exemplary waveform to demonstrate the operation of the wireless implantable GI modulation system is shown in Figure 7.

### 3.4. Preliminary In Vivo Chronic Test

**Figure 8:**
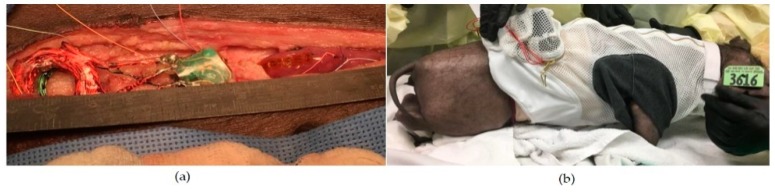
Experiment setup of chronic test. (**a**) EGMD was placed inside a pouch created in the abdominal wall with its electrode sutured on top of the intestine. Note that this deployment of each component of the EGMD helps mitigate signal loss of power and data signals due to tissue absorption, as separation between external and internal coils is merely abdominal-wall thickness, while the electrode array can be extended to access the intestine. Same deployment method is also applicable to future human studies to mitigate the issue of signal loss due to tissue absorption. (**b**) RD was carried by the pig.

A preliminary in vivo chronic study was performed to validate the wireless implantable GI modulation system and the identified stimulation protocol for accelerating intestinal motility. Four juvenile mini Yucatan pigs underwent a laparotomy to receive EGMDs that were sterilized by ethylene oxide. Two of the four pigs were the control group that received the EGMDs without turning on the stimulation, while the other two received active EGMDs delivering the stimulation. The EGMD was placed inside a pouch created in the abdominal wall, and the electrode array was sutured on top of the intestine that was the proximal end of the jejunum identified by the ligament of Treitz (Figure 8a) [11]. Fifteen metal beads were placed inside the intestine, about 5 cm above the segment where the electrode array was sutured. Right after surgery, an X-ray contrast was injected into the pig’s stomach through a gastric tube and X-ray images were acquired. The EGMD was then wirelessly powered and configured through the RD to deliver the stimulation to the electrode that was in the middle column and closest to the tip of the array. Each pig was trained to wear a jacket that carried the RD before the surgery (Figure 8b). The pigs were examined three times using X-ray: right after surgery, one day postoperative, and two days to monitor the transit of the X-ray contrast. 

**Figure 9:**
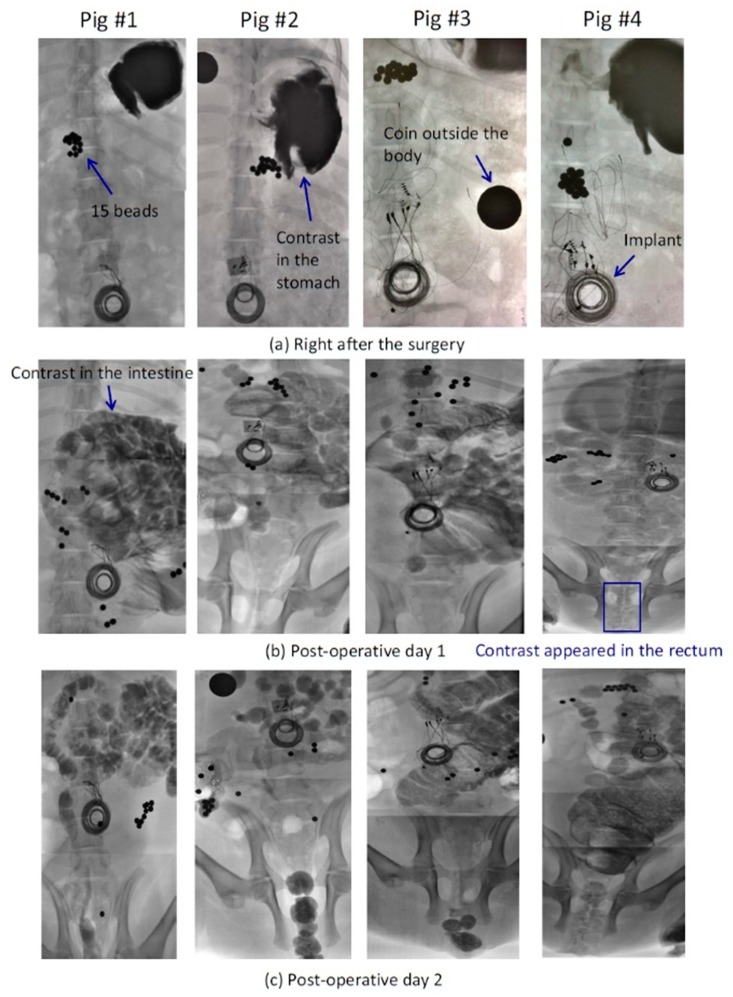
Intestinal transit examined through X-ray photography. Pigs 1 and 2 are control group, while 3 and 4 are the experimental group. (**a**) Fifteen metal beads were inside the intestine, and X-ray contrast was in the pigs’ stomach right after the surgery. (**b**) On postoperative Day 1, the contrast appeared in the rectum in Pig 4 with electrical stimulation, while the contrast had only moved to the intestines in other pigs, suggesting that electrical stimulation likely facilitates intestinal transit. Defecation of feces was also observable in Pig 4 but not in other pigs. (**c**) Contrast moved to the rectum in all pigs on postoperative Day 2. Note that each image is combined with photos taken by the camera as the X-ray machine does not support an image-capture function, and its field of view is not broad enough to show the whole GI tract on the screen.

X-ray results showed that electrical stimulation likely facilitated intestinal transit as the contrast appeared in the rectum in Pig 4 on Day 1, earlier than in the other control pigs (Figure 9). Defecation of feces mixed with the X-ray contrast was also observed in Pig 4 on Day 1, but not clearly in other pigs. However, the movement of metal beads did not show coherent results in X-ray photography. The potential root cause is the small size and slippery surface of the metal beads that make the propelling of the beads inside the GI tract challenging. Although our preliminary data using EGMD show encouraging results, further studies involving more subjects should be conducted.

**Figure 10:**
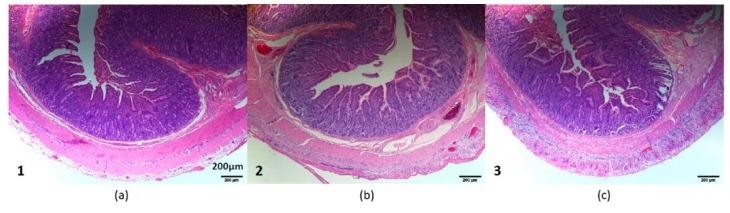
Intestinal-tissue histology. (**a**) Normal jejunum distal to the surgical site (Section 1), (**b**) jejunum that was attached to planar electrode array but did not receive stimulation (Section 2), and (**c**) jejunum that was attached to planar electrode array and received stimulation (Section 3).

Intestinal-tissue histology was also performed to examine for any tissue damage due to stimulation. Three sections of jejunum were compared: normal jejunum distal to the surgical site (Section 1), jejunum that was attached to the planar electrode array but did not receive stimulation (Section 2), and jejunum that was attached to the planar electrode array and received stimulation (Section 3) (Figure 10). Section 2 and Section 3 showed some mild inflammation and hemorrhage in the serosa and longitudinal muscle layer, likely related to the surgical manipulation of these intestinal sections instead of the stimulation. These changes did not appear to be clinically significant. The three different sections appeared otherwise comparable. There was no difference in the villi or crypts seen on these sections, and the submucosa and muscle layers also appeared similar.

## 4. Conclusions

Unlike well-established spinal cord implants and their successful commercialization, there are very few developed GI implantable stimulators, or that have received Food and Drug Administration (FDA) clearance. This might be attributed to the inconclusive GI working mechanism under electrical stimulation and the challenge of developing a versatile implant system with high flexibility. To further the advancement of treating GI dysmotility through electrical neuromodulation (i.e., electroceuticals), we developed a wireless implantable GI modulation system. The system has demonstrated its potential for improving GI motility. The SoC in the implantable EGMD supports wireless power and data telemetries, and a highly programmable stimulator that can generate versatile stimuli. The heterogeneous packaging technique achieves a miniaturized EGMD that is at least an order lighter and 7.7 times smaller than existing commercialized GI implantable stimulators. The RD and the GUI outside the body allow users to wirelessly power and control the EGMD to deliver a tailored stimulation protocol for GI modulation. The system was validated on the bench top and also in preliminary in vivo tests using porcine models. A stimulation protocol for accelerating intestinal transit was obtained and validated in the acute test. The wireless system delivering the proposed protocol likely facilitated GI motility in the preliminary chronic study. In addition, the protocol has shorter pulse width and smaller intensity compared with other works for facilitating GI motility [8,9,10,12,13], which is beneficial for the implantable device in terms of power consumption and stimulation safety. With the versatile stimulation pattern feature, the wireless implantable system not only serves as a research tool for studying an effective stimulation protocol on various GI segments, but also potential personalized medicine that can customize optimal stimulation strategies for each individual. Its versatility and miniaturized size also make it applicable not only to GI electrical stimulation, but also various biomedical applications, including vagus nerve, spinal cord, and nerve regeneration stimulation.

## Figures and Tables

**Table 1:** Summary of measured electrochemical properties of electrode–intestinal fluid and electrode–saline interface.

Electrochemical Property	Saline	Intestinal Fluid
**Cyclic voltammogram**		
Electrochemical window (V)	[−0.9, 1]	[−0.9, 1]
Charge storage capacity (μC)	9.19	10.68
**Electrochemical Impedance Spectroscopy (EIS)**		
Magnitude@ 1 kHz (kΩ)	2.61	6.39
Phase@ 1 kHz (°)	–52.1	–53.9
**Randles cell model characterization**		
R_S_ (kΩ)	1.63	3.69
R_CT_ (kΩ)	2.12	4.7
C_dl_ (nF)	13.73	6.32
